# A novel mutation deep within intron 7 of the *GBA* gene causes Gaucher disease

**DOI:** 10.1002/mgg3.1090

**Published:** 2020-01-14

**Authors:** Anna Malekkou, Ioanna Sevastou, Gavriella Mavrikiou, Theodoros Georgiou, Lluisa Vilageliu, Marina Moraitou, Helen Michelakakis, Chrystalla Prokopiou, Anthi Drousiotou

**Affiliations:** ^1^ Department of Biochemical Genetics The Cyprus Institute of Neurology and Genetics Nicosia Cyprus; ^2^ Cyprus School of Molecular Medicine Nicosia Cyprus; ^3^ Department of Genetics Faculty of Biology Universitat de Barcelona IBUB, CIBERER Barcelona Spain; ^4^ Department of Enzymology and Cellular Function Institute of Child Health Athens Greece; ^5^ Department of Haematology Limassol General Hospital Limassol Cyprus; ^6^ Present address: Reta Lila Weston Institute & Department of Clinical and Movement Neurosciences UCL Queen Square Institute of Neurology London UK

**Keywords:** Cypriot, deep intronic mutation, Gaucher disease, *GBA*, glucocerebrosidase

## Abstract

**Background:**

Mutations in the *GBA* gene that encodes the lysosomal enzyme acid β‐glucocerebrosidase cause Gaucher disease (GD), the most common lysosomal storage disorder. Most of the mutations are missense/nonsense, however, a few splicing mutations within or close to conserved consensus donor or acceptor splice sites have also been described. The aim of the study was to identify the mutation(s) in a Cypriot patient with type I GD.

**Methods:**

The genomic DNA of the proband was screened for nine common mutations using Polymerase chain reaction–restriction fragment length polymorphism (PCR–RFLP) analysis. All exons and exon‐intron boundaries, and the 5’UTR and 3’UTR regions of the *GBA* gene, were investigated by Sanger sequencing. RNA analysis was performed using standard procedures, and the abnormal transcript was further cloned into pGEM‐T‐Easy plasmid vector and sequenced. The relevant intronic region was further sequenced by the Sanger method to identify the genetic variant.

**Results:**

A novel point mutation, g.12599C > A (c.999 + 242C > A), was detected deep in intron 7 of the *GBA* gene. This type of mutation has been previously described for other diseases but this is the first time, as far as we know, that it is described for GD. This mutation creates a new donor splice site leading to aberrant splicing and resulting in the insertion of the first 239nt of intron 7 as a pseudoexon in the mRNA, creating a premature stop codon.

**Conclusion:**

This study expands the mutation spectrum of GD and highlights the importance of RNA sequencing for the molecular diagnosis of patients bearing mutations in nonexonic regions.

## Graphical abstract

This study identified a novel mutation deep in intron 7 of the *GBA* gene. This mutation creates a new splice donor site leading to the insertion of the first 239 nucleotides of intron 7 in mRNA, resulting in a premature stop codon.

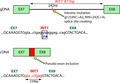

## 1 INTRODUCTION

Gaucher disease (GD) is the most common lysosomal storage disorder. GD follows an autosomal recessive mode of inheritance and is commonly due to loss‐of‐function mutations in the *GBA* gene (OMIM: 606,463), encoding glucocerebrosidase (GCase, E.C.3.2.1.45), and more rarely due to mutations in the *PSAP* gene, encoding saposin C. GCase is responsible for the degradation of glucosylceramide (GluCer) to glucose and ceramide inside the lysosomes (Brady, Kanfer, Bradley, & Shapiro, 1966). GCase deficiency results in lysosomal accumulation of GluCer in macrophages, known as ‘Gaucher cells’, which are key players in the pathophysiology of the disease. Activated macrophages secrete chitotriosidase, which reaches very high values in plasma and is used as a marker for disease progression and for monitoring response to treatment (Hollak, van Weely, van Oers, & Aerts, 1994). A common polymorphism in exon 10 of the *CHIT1* gene causes deficiency of chitotriosidase, with no clinical consequences. Gaucher patients homozygous for this polymorphism are monitored using other biomarkers such as PARC‐CCL18.

GD patients are generally classified into three distinct types based upon the absence or presence of neurological symptoms (Sidransky, 2012); type 1 (nonneuronopathic), type 2 (acute neuronopathic), and type 3 (subacute neuronopathic). The most common type is the nonneuronopathic type 1 (OMIM#230800) which presents with systemic manifestations such as hepatosplenomegaly, anemia, thrombocytopenia, and bone abnormalities. Type 2 (OMIM#230900) and 3 (OMIM#2301000) GD are characterized by manifestations of the central nervous system, with type 2 being more severe, with life‐threatening neurological disease in infancy.

The *GBA* gene is located on chromosome 1q21 and consists of 11 exons and 10 introns spanning a sequence of 7.6 Kb (Ginns et al., 1985). A highly homologous pseudogene (*GBAP*), which shares 96% coding sequence similarity with *GBA*, is located 16 Kb downstream of the *GBA* gene (Horowitz et al., 1989). The *GBAP* gene contributes significantly to the generation of mutations in the *GBA* gene due to gene‐pseudogene rearrangements (Tsuji et al., 1987), something that complicates the molecular diagnosis for GD. More than 400 different mutations have been identified so far throughout the *GBA* gene, including point mutations, insertions, deletions, splice‐site mutations, and “complex mutations” due to recombination events of the *GBA* gene and the *GBAP* pseudogene (The Human Gene Mutation Database, http://www.hgmd.org). DNA analysis in Gaucher patients usually starts by screening for the common mutations in the population, and if negative this is followed by Sanger sequencing. More recently, a sequencing method using long reads on the Oxford Nanopore minION platform was shown to be able to detect most disease causing variants with the added advantages of phasing and intronic analysis (Leija‐Salazar et al., 2019).

GD is rare in Cyprus with only three unrelated patients having been diagnosed with type I GD in the last 30 years. Two patients were found to be compound heterozygotes, with the genotype N370S/L444P. The molecular investigation of the third patient was the subject of this study.

## 2 MATERIALS AND METHODS

### 2.1 Ethical compliance

This work is covered by the code of Ethics of the Cyprus Institute of Neurology and Genetics. All persons who were included in this study gave their informed consent.

### 2.2 Case Report

The proband is a Greek Cypriot lady who was referred to the Hematology clinic at the age of 42, as she was found to have hepatosplenomegaly (liver 8 cm, spleen 10 cm). Her hematological parameters were within normal ranges except for elevated acid phosphatase (20.6 mg/dl, normal range: 1–4.7 mg/dl), thrombocytopenia (platelets 8 × 10^4^/mm^3^) and hypergammaglobulinemia. Μyelogram and bone marrow biopsy showed the characteristic “Gaucher cells". Diagnosis was confirmed by measuring GCase activity in isolated leukocytes. The proband also had a very low value of plasma chitotriosidase (chito) and was found to be homozygous for the 24 bp duplication of the *CHIT1* gene. The proband did not develop any neurological symptoms and was classified as Type 1 GD. The patient started enzyme replacement therapy (ERT) at the age of 53 with a biweekly dosage of 20 IU/kg. After 1 year of treatment, hemoglobin increased from 11.5 g/dl to 12.4 g/dl, and platelets increased from 47,000/mm^3^ to 54,000/mm^3^. The size of the liver and spleen was reduced by 30% and 40% respectively. The patient continues to receive ERT without any side effects. Treatment is monitored by measuring the PARC‐CCL18 biomarker in plasma.

### 2.3 Enzyme measurements

EDTA plasma and a lysate of isolated white blood cells from peripheral blood were used for the determination of chitotriosidase and GCase activities respectively. The activities were measured using artificial fluorescent substrates as previously described (Mavrikiou, Petrou, Georgiou, & Drousiotou, 2016; Wenger, Clark, Sattler, & Wharton, 1978).

### 2.4 DNA and RNA analysis

Details of methods and kits used can be found in Tables S1‐S4 in Appendix S1.

Genomic DNA from the proband, family members and control individuals was extracted from peripheral blood. Total RNA was isolated from the patient's whole blood and control individuals, and cDNA was synthesized from 1 μg of total RNA. PCR amplification was conducted with the DNAs and cDNAs that were obtained. For restriction enzyme analysis, specific DNA fragments of the *GBA* gene were amplified by PCR, using specific primers that prevent the amplification of *GBAP*, followed by the appropriate restriction enzyme digestion (Table S2 in Appendix S1). For cloning, the desired amplified PCR fragments were purified from agarose gel and subcloned into pGEM‐T‐Easy plasmid vector (Promega). Randomly selected individual clones were grown in LB medium at 37°C overnight and the plasmid DNAs were isolated. The amplified PCR products and the cDNA plasmid inserts were bidirectionally sequenced using specific primers (Table S3 in Appendix S1). The resultant sequences were aligned and compared to the *GBA* genomic (NG_009783.1) or cDNA (NM‐000157.4) sequences from Genbank reference sequence (NC_000001.11) using the NCBI Blast tool. Bioinformatics prediction analysis for possible changes in splice sites was performed using the NNSplice program from the Berkeley Drosophila Genome Project (http://fruitfly.org/seq_tools/splice.html).

RFLP analysis was performed to confirm the mutation. Nested PCR was performed using EX7_F and N370S_R followed by EX7_F and IN7_R primers (Table S4 in Appendix S1). The product was digested with the Hpy188III restriction endonuclease for 2 hours at 37°C and this was followed by electrophoresis in an agarose gel. RFLP analysis was also used to screen for the mutation in 100 samples from normal individuals.

## 3 RESULTS

### 3.1 Enzyme results

**Figure 1:**
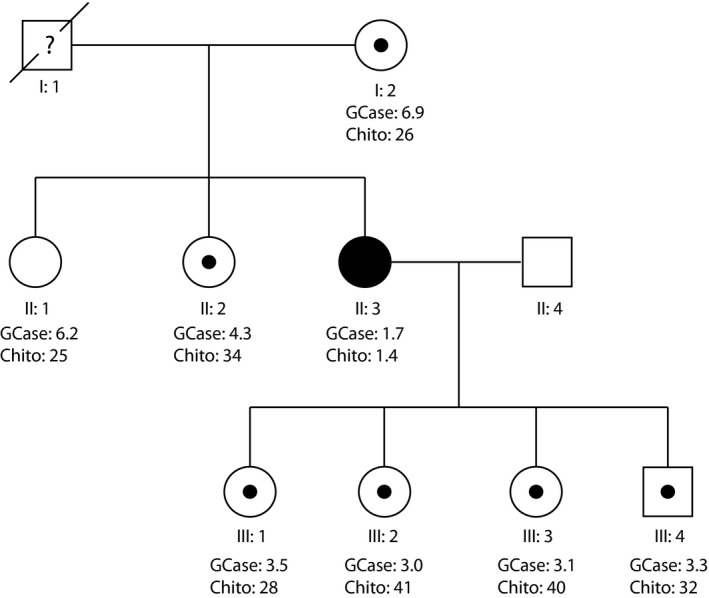
Family pedigree. Black symbols represent affected persons and symbols with a dot, carriers. The levels of GCase and chitotriosidase activity can be seen underneath each individual. Normal ranges: GCase: 5.4–16.8 nmol mg protein^-1^ hr^-1^, Chito: 9.5–44 nmol ml^‐1^ hr^‐1^

The diagnosis of GD in the proband was confirmed by the measurement of GCase activity which was found to be 1.7 nmol mg^‐1^ hr^‐1^ (normal range 5.4–16.8 nmol mg^‐1^ hr^‐1^). Plasma chitotriosidase activity was also very low, 1.4 nmol ml^‐1^ hr^‐1^ (normal range 9.5‐44 nmol ml^‐1^ hr^‐1^), and the patient was found to be homozygous for the 24 bp duplication polymorphism (c.1049_1072dup24) in exon 10 of the *CHIT1* gene. For this reason, the treatment of this patient is monitored by measuring the PARC‐CCL18 biomarker in plasma. The values for the GCase and chitotriosidase activity for the proband and other family members are shown in Figure 1.

### 3.2 Screening for common mutations

The genomic DNA of the patient was first screened for nine common mutations in the *GBA* gene: N370S, L444P, D409H, R463C, 55bpdel, IVS10‐1G > A, IVS6‐2A > G, R120W, and Y108C, by PCR–RFLP analysis. No mutation was detected.

### 3.3 Sanger sequencing

We then proceeded to sequence all exons of the *GBA* gene, including exon–intron boundaries and the 5’UTR and 3’UTR regions. No mutation was identified.

### 3.4 cDNA analysis

**Figure 2:**
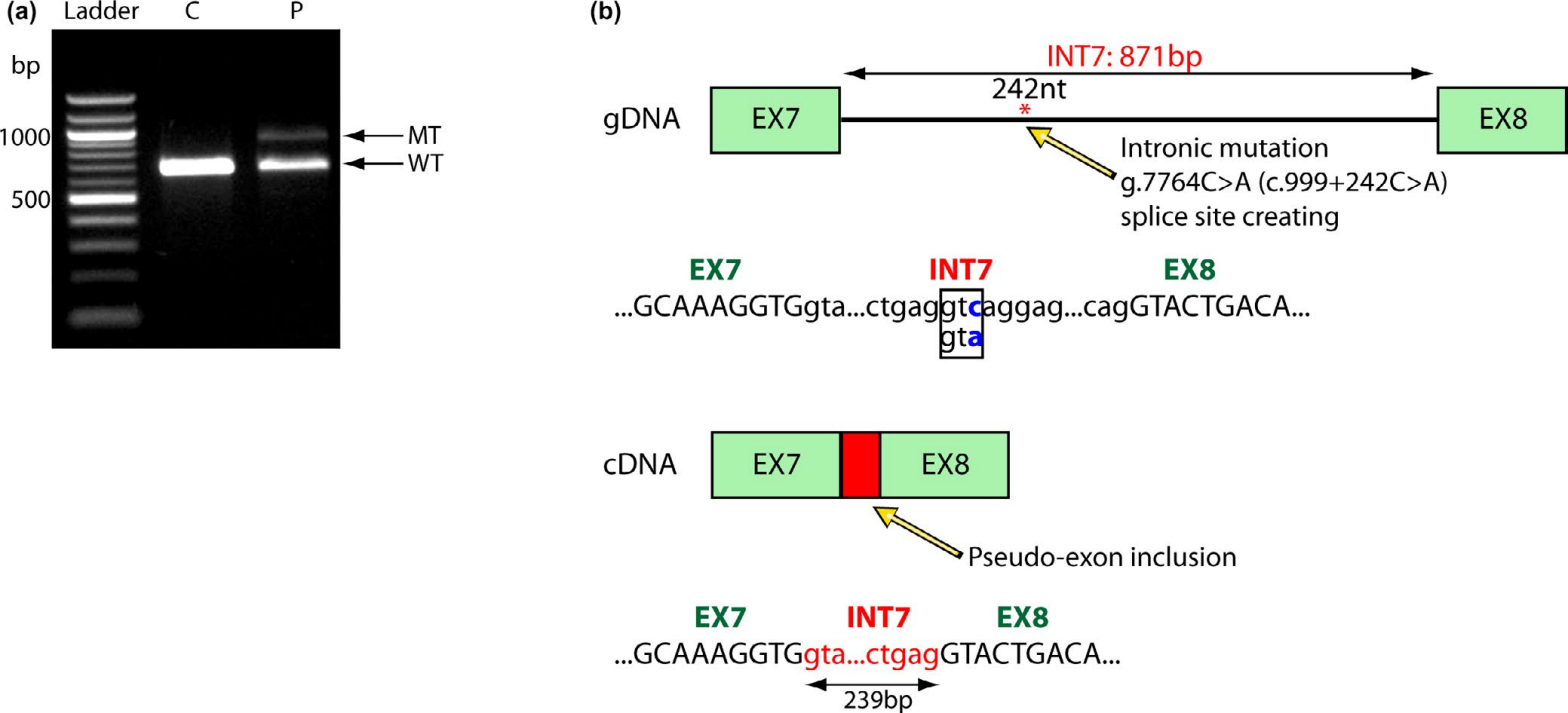
(a) cDNA amplicons of patient (P) and healthy control (C) showed an additional transcript at higher molecular weight (arrow) in patient sample. (b) Schematic representation of the gDNA and cDNA sequences of the *GBA* gene from exon 7 to exon 8 (Ex7‐Ex8), showing the activation of a cryptic splice site (*), 242 bp upstream of exon 7 due to C > A substitution, leading to pseudoexon inclusion. Exonic and intronic sequences are written with capital and small letters, respectively. The GenBank reference sequence of *GBA* gene is NG_009783.1 (NC_000001.11)

To determine whether aberrant RNA splicing occurred, total RNA was isolated and the cDNA was generated by reverse transcriptase. Different PCR amplicons of cDNA were generated and then bidirectionally sequenced. Gel electrophoresis of the cDNA amplicon spanning exons 6–10 yielded an additional band of higher molecular weight (Figure 2a). The DNA corresponding to the two bands was separately extracted from the gel and cloned into a pGEM‐TEasy vector and the clones were sequenced. The clone corresponding to the lower molecular weight fragment was identical to the normal transcript sequence (WT). However, the higher molecular weight transcript revealed an insertion of 239 bp between exons 7 and 8. After blast analysis of this sequence, this insertion was found to be identical with the first 239nt of intron 7 (Figure 2b). This finding suggested the presence of a genomic mutation leading to alternative splicing. Subsequent amplification and sequencing of the gDNA region spanning exon 7 to exon 8 revealed a novel mutation at nucleotide position g.12599 (Chr1: 155,237,099, GRCh38.p13), deep within intron 7, resulting in a C to A substitution. The proband was homozygous for this mutation. In silico analysis using the NNsplice program, searching for potential splice site sequences in the amplified PCR product (spanning exon 7 to exon 8 including intron 7), confirmed the creation of a new donor splice site (score 1.0). The novel mutation creates a new splice donor site leading to the insertion of the first 239 nucleotides of intron 7 in mRNA, resulting in a premature stop codon.

**Figure 3:**
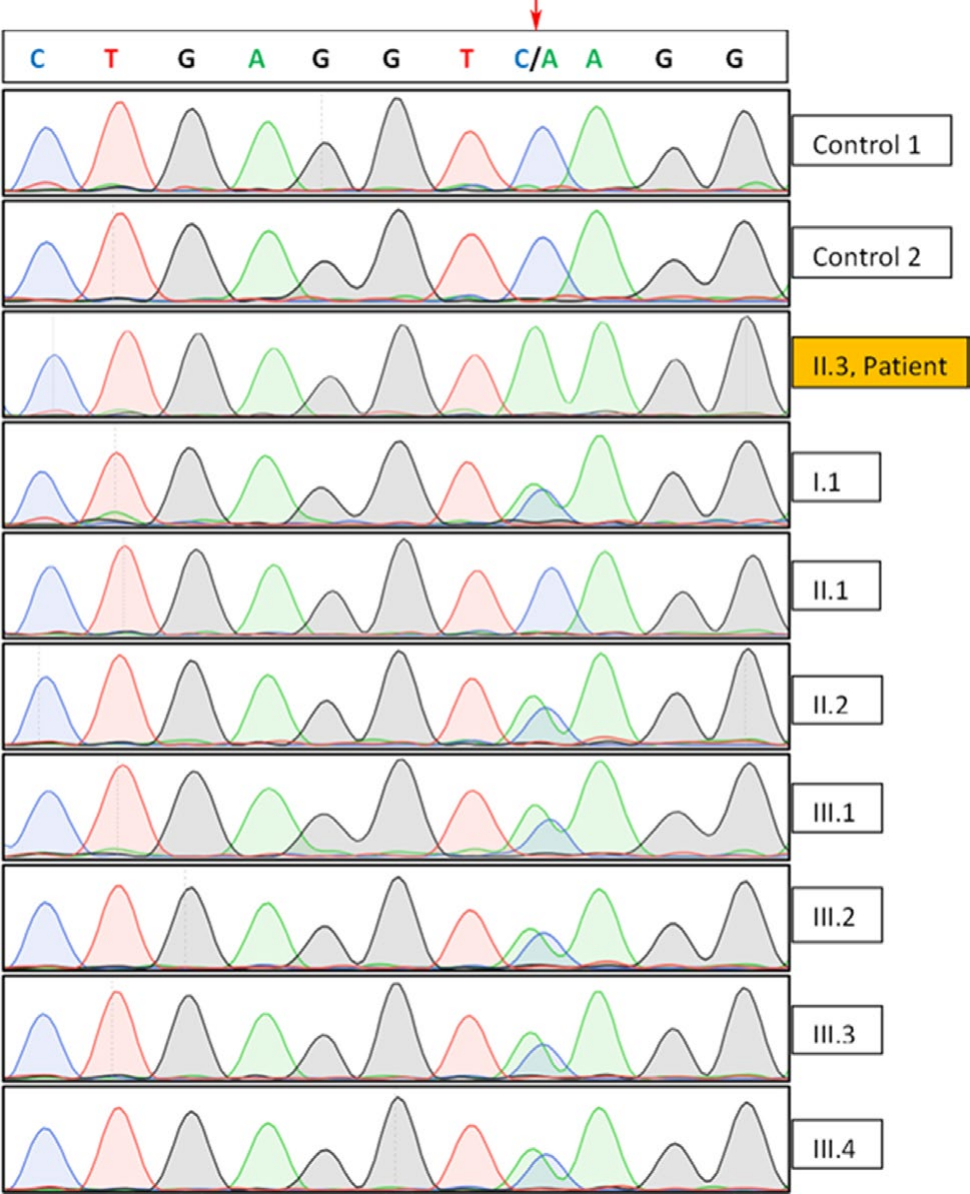
Electropherogram of Sanger sequence analysis of PCR amplicons of Ex7‐Ex8. The patient (II.3) carries a homozygous mutation g.12599C > A (arrow), which is absent from controls. The mutation was also found in the heterozygous state in the patient's mother (I.1), one sister (II.2), and four children (III.1‐III.4), as indicated by a double peak depicting the normal and the mutant sequence. The GenBank reference sequence of *GBA* gene is NG_009783.1 (NC_000001.11)

The mutation was also found in the heterozygous state in one of the parents (the other parent is not alive), one sister, and four children of the proband, and was absent in controls (Figure 3). It is noteworthy that the parents of the proband originate from the same village.

### 3.5 RFLP analysis

The mutation g.12599C > A abolishes a cleavage site for the Hpy188III restriction endonuclease: the patient showed only a single band at 753 bp, while the normal control showed two DNA fragments at 418 bp and 335 bp. RFLP analysis was used to screen for the mutation in 100 normal Greek Cypriots, originating from all major geographic areas of Cyprus. All samples were negative for the mutation.

## 4 DISCUSSION

Approximately 10% of about 80,000 mutations reported in the human gene mutation database (HGMD) affect splicing. About 600 mutations that affect the pre‐mRNA splicing process have been documented in patients with lysosomal storage disorders (Dardis & Buratti, 2018). Most of these splicing mutations occur within or close to conserved consensus donor (+1/+2) or acceptor (‐1/‐2) splice sites (Krawczak, Reiss, & Cooper, 1992) and can be detected by Sanger sequencing of PCR amplicons of exon and exon–intron boundary regions. Alterations in splice sites usually cause aberrant splicing due to disruption of recognition and interaction of the spliceosome with these sequences during mRNA splicing. Splicing mutations have also been reported in a variety of other positions that are fairly distant from splice sites (Lewandowska, 2013), such as point mutations within the branch point sequence. Instances of pathogenic mutations in deep intronic regions, other than branch point sites, have been published. Usually, these deep intronic mutations are located more than 100 bp away from exon–intron boundaries and are associated with aberrant splicing, and most commonly, with pseudoexon inclusion due to activation of a cryptic splice site. Deep intronic mutations leading to pseudoexon inclusion have been documented in multiple diseases with increasing frequency: in patients with neurofibromatosis (Cunha et al., 2016), Duchenne muscular dystrophy (Trabelsi et al., 2014), HPRT deficiency (Corrigan, Arenas, Escuredo, Fairbanks, & Marinaki, 2011), as well as several hereditary tumor syndromes, like melanoma (Harland, Mistry, Bishop, & Bishop, 2001), retinoblastoma (Dehainault et al., 2007), and breast cancer (Anczukow et al., 2012).

In the present study we have identified a novel mutation deep in intron 7 of the *GBA* gene in a Cypriot patient with type 1 GD. This is the first time that this type of mutation is described for GD. This mutation creates a cryptic splice donor site, leading to the creation of an aberrant transcript with an insertion of the first 239nt of intron 7 between exons 7 and 8. This transcript contains a 239 bp pseudoexon with a premature stop codon that may cause mRNA degradation due nonsense mediated mRNA decay (NMD) (Maquat, 2004). This may explain why the % of the mutant transcript in the patient is relatively small compared to the WT transcript. Moreover, the c.999 + 242C > A mutation does not abolish the generation of WT mRNA, as it was found to be present in our patient, who is homozygous for this mutation; this may explain the relatively mild phenotype of the patient and the late onset of the disease. We have also developed an appropriate screening test, that utilizes Hpy188III restriction endonuclease digestion, for confirmation and for future efficient identification of the g.12599C > A (c.999 + 242C > A) mutation.

Mutations in the *GBA* gene have been shown to be associated with an increased risk for developing Parkinson's disease, with the heterozygote carriers having about a fivefold higher risk (Gan‐Or et al., 2015). In this respect it is interesting to note that the mother of our proband developed Parkinson's disease, raising the possibility that the g.12599C > A (c.999 + 242C > A) mutation might act as a risk factor for Parkinson's disease.

In conclusion, this study expands the mutation spectrum of GD and highlights the importance of RNA sequencing for the molecular diagnosis of patients bearing mutations in nonexonic regions.

## CONFLICT OF INTEREST

The authors declare no conflicts of interest.

## AUTHOR CONTRIBUTIONS

Anna Malekkou contributed to the design of the study, the execution of most of the experiments, the interpretation of the results, and the writing of the manuscript. Ioanna Sevastou, Gavriella Mavrikiou, Theodoros Georgiou, and Lluisa Vilageliu contributed to the experimental work and the interpretation of the results. Marina Moraitou and Eleni Michelakakis contributed to the experimental work, the interpretation of the results, and writing of the manuscript. Chrystalla Prokopiou provided the clinical information on the patient. Anthi Drousiotou designed and supervised the study and contributed to the writing, revising, and editing of the manuscript. All authors approved the final version of the manuscript.

## Data Availability

The variant of this study has been submitted to ClinVar database (http://www.ncbi.nlm.nih.gov/clinvar/), reference accession number SCV00930452. Other data are available from the corresponding author upon request.
