# Supplementary material for: A novel mutation deep within intron 7 of the GBA gene causes Gaucher disease
Source: Mol Genet Genomic Med. 2020 Jan 14;8(3):e1090. doi: 10.1002/mgg3.1090 (PMC7057115; doi:10.1002/mgg3.1090)
Supplement: Supplementary file 1 [file MGG3-8-e1090-s001.docx]

**Appendices**

**Table S1: Kits and enzymes that were used in this study**

| **Procedure** | **Kit** | **Company** |
| --- | --- | --- |
| DNA extraction from blood | Blood DNA Extraction Kit | Qiagen |
| RNA extraction from blood | NucleoSpin RNA Blood kit | Macherey-Nagel |
| cDNA synthesis | Protoscript® M-MuLV II First Strand cDNA Synthesis Kit | New England Biolabs |
| PCR amplification | Gold Taq DNA polymerase | Invitrogen |
| Restriction Fragment Length Polymorphism (PCR-RFLP) screening | Restriction enzymes | New England Biolabs |
| DNA agarose gel purification | NucleoSpin® Gel and PCR clean-up kit | Macherey-Nagel |
| Plasmid isolation | NucleoSpin® Plasmid mini kit | Macherey-Nagel |
| Sequencing | BigDye® Terminator v1.1 Cycle Sequencing kit | Applied Biosystems |
|  | Gel Filtration Cartridges |  |

**Table S2: Primers and enzymes that were used for RLFP analysis of common mutations in *GBA* gene**

| **Mutations** | **Forward primers** | **Reverse primers** | **PCR product size (bp)** | **Restriction enzymes** |
| --- | --- | --- | --- | --- |
| N370S | GCCTTTGTCCTTACCCTCG | GACAAAGTTACGCACCCA | 869 | XhoI |
| L444P | CGTAACTTTGTCGACAGT | TCCCAGACCTCACCATTG | 600 | NciI |
| D409H | AACCATGATTCCCTATCTTC | GCTCCCTCGTGGTGTAGAGT | 524 | StyI |
| R463C | CGTAACTTTGTCGACAGTCC | TGCTGTGCCCTCTTTAGTCA | 869 | MspI |
| 55bpdel | AACCATGATTCCCTATCTTC | GCTCCCTCGTGGTGTAGAGT | 524 | Lower MW band on 2% agarose gel |
| IVS10-1G>A | CGTAACTTTGTCGACAGTCC | TGCTGTGCCCTCTTTAGTCA | 869 | MspI/HphI |
| IVS6-2A>G | TTGGCCGGATCATTCATGAC | CTAGGTCACGGGCAATGAAG | 183 | MspI |
| R120W | GCGAACTCCTGACCTCGTGATCTG | CTTGAGCTTGGTATCTTCCTCTGG | 499 | NciI |
| Y108C | TGGGTACTGATACCCTTATT | TCAATGGCTCTATGTCATCT | 223 | RsaI |

**Table S3: Primers that were used for the PCR amplification and Sanger sequencing of the coding exons and UTR**

| **Name of primers**† | **Sequence of primers** |
| --- | --- |
| GBA1_5UTRF | TCTTCATCTAATGACCCTGA |
| GBA1_EX4F | GCAGCCAGAACAGAAGTTCC |
| GBA1_EX5F | GAATCGGATATAACATCATCC |
| GBA1_EX6F | GACCTGGGCCAGATACTTTG |
| GBA1_EX7F | CTTCTGCTGGGCTGTTGAGT |
| GBA1_EX5R | GGAAATCATCAGGGGTGTC |
| GBA1_EX7R | GAAGGGGTATCCACTCAACA |
| GBA1_IN7R | TGTGTGTATGTATGTGTGTGTGTG |
| GBA1_EX10R | GGAGCCCTCAGGAATGAACT |
| GBA1_RV3UTR | AAGAGCTGCCATTTTTCCTG |

†F denotes forward primers, R reverse primers, EX exon, IN intron

**Table S4: Primers that were used for the RFLP analysis of the g.12599C>A (c.999+242C>A) mutation**

| **Name of Primer** | **Sequence** |
| --- | --- |
| EX7F | CTTCTGCTGGGCTGTTGAGT |
| N370S | GACAAAGTTACGCACCCAA |
| IN7R | TGTGTGTATGTATGTGTGTGTGTG |
